# Influence of Precursors on Physical Characteristics of MoS_2_ and Their Correlation with Potential Electrochemical Applications

**DOI:** 10.3390/ma18092111

**Published:** 2025-05-04

**Authors:** Cătălin Alexandru Sălăgean, Liviu Cosmin Coteț, Monica Baia, Carmen Ioana Fort, Graziella Liana Turdean, Lucian Barbu-Tudoran, Mihaela Diana Lazar, Lucian Baia

**Affiliations:** 1 Laboratory for Advanced Materials and Applied Technologies, Institute for Research, Development and Innovation in Applied Natural Sciences, “Babes-Bolyai” University, Fantanele 30, 00294 Cluj-Napoca, Romania; catalin.salagean@ubbcluj.ro (C.A.S.); monica.baia@ubbcluj.ro (M.B.); ioana.fort@ubbcluj.ro (C.I.F.); 2 Doctoral School of Physics, Faculty of Physics, “Babes-Bolyai” University, M. Kogălniceanu 1, 400084 Cluj-Napoca, Romania; 3 Research Center of Electrochemistry and Non-Conventional Materials, Department of Chemical Engineering, Faculty of Chemistry and Chemical Engineering, “Babes-Bolyai” University, Arany Janos 11, 400028 Cluj-Napoca, Romania; graziella.turdean@ubbcluj.ro; 4 Electron Microscopy Center, Faculty of Biology and Geology, “Babes-Bolyai” University, 5-7 Clinicilor St., 400006 Cluj-Napoca, Romania; lucian.barbu@ubbcluj.ro; 5 National Institute for Research and Development of Isotopic and Molecular Technologies, 67-103 Donat St., 400293 Cluj-Napoca, Romania; diana.lazar@itim-cj.ro

**Keywords:** molybdenum disulfide, characterization, precursors

## Abstract

MoS_2_, a key material for supercapacitors, batteries, photovoltaics, catalysis, and sensing applications, was synthesized using the hydrothermal method. Different precursors such as molybdenum sources (ammonium heptamolybdate tetrahydrate ((NH_4_)_6_Mo_7_O_24_·4H_2_O) and sodium molybdate hydrate (Na_2_MoO_4_·2H_2_O)) combined with L-cysteine, thiourea, and thioacetamide, as the sulfur source, were involved. The obtained samples were morphologically and structurally characterized by X-ray diffraction, Raman spectroscopy, N_2_ adsorption/desorption measurements, and Scanning Electron Microscopy with Energy-Dispersive X-ray Spectroscopy (SEM–EDX). Electrochemical impedance spectroscopy was involved in MoS_2_ characterization as electrode materials. The objective of this study was to ascertain the impact of precursor combinations on the morphological, structural, and electrochemical characteristics of MoS_2_. A thorough examination of the empirical data revealed that the MoS_2_ compounds, which were synthesized using thiourea as the sulfur source, exhibited a more pronounced flower-like morphology, increased crystallite size, and enhanced electrochemical properties with potential electrochemical applications.

## 1. Introduction

Energy is a key research topic today, driven by the need for efficient storage systems and eco-friendly production [1]. With growing consumption and technological advances, the shift from polluting fossil fuels [2] to sustainable sources like photovoltaics [3,4], wind power [5], or green chemical routes such as hydrogen evolution reaction (HER) for fuel cells is essential. Ongoing progress in energy production requires equally advanced storage solutions, prioritizing materials and designs to meet modern demands for higher energy density and power. While batteries offer high energy density, their relatively lower power density and longer charging times limit their application in high-power-demand scenarios. This inherent limitation restricts their applicability in scenarios where high power is requisite and expeditious energy transfer or storage is imperative. In this context, supercapacitors can serve as primary storage units, addressing challenges related to charging times and power output. When it comes to this aspect, batteries have traditionally been the primary solution for addressing storage challenges, thanks to their high energy density [6]. However, modern technologies now integrate supercapacitors to enhance power density, extend lifespan, and reduce charging times, better meeting contemporary demands. Due to their simple operating mechanism based on electrical double layer (EDL) or pseudo-capacitance, along with high charge/discharge rates and long cycle life, supercapacitors present a promising energy storage system [7]. In this case, supercapacitors can be considered superior to batteries simply because charging in supercapacitors is not limited by the diffusion of ions in the active material, so they could complement or even replace batteries when high power is needed to be delivered [8]. It is important to note the use of transition-metal dichalcogenides (TMDCs) because of their unique physical and chemical features reported in recent years, mainly due to the large specific surface area and great redox chemistry that they undergo to show significant potential in building better supercapacitors [1]. In this respect, many research groups [9,10,11] focused on studying relatively novel materials based on MoS_2_ that can be used in building supercapacitors.

Although hydrothermal synthesis of MoS_2_ has advanced significantly, key gaps remain. The roles of intermediate species, reaction pathways, and precursor influence on nucleation and growth are not well understood. Additionally, the stability and coexistence of 1T and 2H phases require further study for electrochemical optimization. Limited correlations between morpho-structural and electrical properties across different synthesis routes also hinder progress. Addressing these issues is crucial for fully harnessing MoS_2_ in advanced energy and sensing applications. Therefore, optimizing the synthesis of MoS_2_ is crucial for enhancing conductivity, capacitance, and surface area, ultimately improving the material’s overall performance in supercapacitor applications. As previously mentioned, the crystalline phase is a crucial factor influencing the material’s performance. Naturally, MoS_2_ presents semiconductive properties and is thermodynamically stable, while the 1T-metallic phase exhibits promising electrochemical properties, such as better hydrophilicity and high electrical conductivity (i.e., 107 times higher than the semiconductive 2H phase) [12]. By surveying the literature, it can be observed that research groups tried to optimize MoS_2_ properties in various applications by varying the reaction time, temperature, or reducing agent. Other studies were focused on precursors: molybdate ((NH_4_)_6_Mo_7_O_24_·4H_2_O) and thioacetamide (C_2_H_5_NS) [13,14,15], L-cysteine [16], or thiourea (CH_4_N_2_S) as a sulfur source [17,18,19].

A promising technique for creating two-dimensional transition-metal dichalcogenide (TMDC) MoS_2_ nanostructures is hydrothermal synthesis. Such obtained nanostructures have remarkable properties, such as precise control over morphology, crystallinity, and surface properties, including layered structure, high electrical conductivity, and excellent catalytic activity [20,21,22]. These properties can be exploited in a large variety of versatile electrochemical devices involving supercapacitors and electrochemical catalysts [23].

Moreover, MoS_2_ hydrothermal synthesized material has been used in the development of sensing devices for the detection of gases, chemicals, and biomolecules. Thus, advanced sensors with improved selectivity and sensitivity were developed due to their unique electronic properties, high surface-to-volume ratio and chemical stability, and applied in environmental monitoring, healthcare diagnostics, and different industrial applications.

As a catalyst, MoS_2_ exhibits high catalytic activity in various electrochemical reactions, excellent stability, and low overpotential for the hydrogen evolution reaction (HER), making it a promising candidate for efficient and sustainable hydrogen production [24]. Furthermore, MoS_2_-based catalysts have shown potential in other electrochemical reactions, including oxygen reduction reaction (ORR), hydrogen oxidation reaction (HOR), and nitrogen reduction reaction (NRR) [25,26], paving the way for their use in fuel cells, water electrolysis, and electrochemical sensing platforms.

To the best of our knowledge, no comparative study has yet been conducted on the synthesis process using different precursors (such as Mo and sulfur sources) under the same experimental conditions. Therefore, this study is focused in its first stage on the hydrothermal synthesis of MoS_2_ using two of the most used molybdenum sources (ammonium heptamolybdate tetrahydrate ((NH_4_)_6_Mo_7_O_24_·4H_2_O) and sodium molybdate hydrate (Na_2_MoO_4_·2H_2_O)) combined with L-cysteine, thiourea, and thioacetamide, as sulfur source. Afterward, the six hydrothermally obtained samples were characterized in terms of structure (X-ray diffraction—XRD), surface (N_2_ adsorption–desorption measurements), morphology (Scanning Electron Microscopy with Energy-Dispersive X-ray Spectroscopy—SEM–EDX) and electrochemical properties (electrochemical impedance spectroscopy, EIS). In this context, our interest was focused on understanding the influence of different commonly used precursors in the synthesis of MoS_2_ on its morphological and structural properties from the perspective of its electrochemical EIS performance. The ultimate goal of this approach is to select the most suitable synthesis precursors for potential electrochemical- applications, such as supercapacitors, HER or ORR catalysts, and sensing materials.

## 2. Materials and Methods

### 2.1. MoS_2_ Synthesis

In the synthesis of MoS_2_ samples, ammonium molybdate ((NH_4_)_6_Mo_7_O_24_·4H_2_O) and sodium molybdate (Na_2_MoO_4_·2H_2_O) were used as molybdenum precursors, while for the sulfur source L-cysteine (C_3_H_7_NO_2_S), thioacetamide (C_2_H_5_NS), and thiourea (CH_4_N_2_S) compounds were used. Various combinations of precursors are used to prepare MoS_2_ samples. In a typical reaction, 42 mM of molybdenum precursors and 840 mM of sulfur sources were mixed with 60 mL MilliQ water (40% reactor filling volume) and were vigorously stirred for 60 min. The higher concentration of sulfur precursor was mainly due to the improved crystallinity of MoS_2_. After that, the solutions were transferred in a PPL-lined stainless-steel autoclave for 24 h at 200 °C. After the reactor had naturally cooled down to room temperature, the black precipitate was collected and washed thoroughly using water and absolute ethanol and dried at 60 °C for 24 h. The samples derived from different combinations of Mo and S precursors are labeled as shown in Table 1.

### 2.2. Characterization

XRD diffractograms were obtained by using a Shimadzu XRD-6000 diffractometer (Kyoto, Japan), operating with CuK α radiation (λ = 1.54 nm) and a Ni filter. The diffraction patterns were recorded in the 2θ range between 5° and 60° with a scan speed of 2°/min.

Raman spectra were recorded by using a Renishaw InVia Reflex Raman spectrometer (Wotton-under-Edge, UK) equipped with an air-cooled RenCam CCD detector. A 532 nm laser line with a power of 200 mW was employed as the excitation source.

Scanning Electron Microscopy (SEM) measurements were performed with a Hitachi SU8230 Scanning Electron Microscope (SEM) (Hitachi Company, Tokyo, Japan) equipped with the Energy-Dispersive Spectroscopy (EDS) detector X-Max 1160 EDX (Oxford Instruments, Oxford, UK). The investigation was effectuated in the high vacuum mode at an acceleration voltage of 30 kV.

Nitrogen adsorption–desorption isotherms were recorded at −196 °C using the Sorptomatic 1990 apparatus (Thermo Electron, Milan, Italy). Total surface area (St) and porosity parameters (pore volume Vp and mean size Dm) were calculated from the isotherms using the BET model and Dollimore–Heal method, respectively. Before measurements, the samples were degassed in a vacuum for 4 h at 125 °C. Samples B and F could not be measured due to low surface area for sample B (below 1 m^2^/g) and instability on the degassing step for sample F.

### 2.3. Electrode Preparation

For the electrochemical characterization of the six MoS_2_-based materials (A, B, C, D, E, and F), a suspension of these materials in chitosan was prepared. Thus, 1 mg/mL chitosan in 0.1 M acetic acid was prepared. At this solution, 1 mg of each MoS_2_-based material was added, leading to six suspensions, which were sonicated for 1 h. A volume of 5 µL of the suspension was dropped on the glassy carbon electrode clean surface (GCE, from Radiometer, inner diameter of 3 mm). Therefore, for each electrode, 5 × 10^−6^ g was used. The modified electrode was allowed to dry for 24 h at room temperature until total evaporation of the solvent.

### 2.4. Electrochemical Measurements

The electrochemical impedance spectroscopy (EIS) measurements were realized by using a PC-controlled potentiostat (AUTOLAB PGSTAT302N EcoChemie, Utrecht, The Netherlands) with a three-electrode arrangement cell at room temperature. The modified GC/ChMoS_2_ (MoS_2_ = A, B, C, D, E, and F) electrodes were the working electrodes, Ag/AgCl in saturated KCl solution (from Radiometer, Copenhagen, Denmark) was the reference electrode, and a Pt wire was the counter electrode.

The electron transfer abilities at the modified GC/ChMoS_2_ (MoS_2_ = A, B, C, D, E, and F) electrodes surface was realized at the open circuit potential in 0.1 M KCl containing 5 mM K_4_[Fe(CN)_6_]/K_3_[Fe(CN)_6_], at frequency values from 10^4^ Hz to 10^−2^ Hz. The experimental results were fitted with a modified Randless equivalent circuit.

## 3. Results and Discussion

### 3.1. Morphological and Structural Characterization

X-ray diffraction analysis can be considered a powerful tool in the study of nanomaterials due to its capability to determine the crystalline structure of materials and their oriented planes. For MoS_2_, the diffraction peaks that occur at 2θ values of 14°, 33°, and 58° can be attributed to (002), (100), and (110) planes [27]. Additionally, it has been reported that XRD patterns of MoS_2_ can develop peaks around 39°, corresponding to the (103) plane. However, some MoS_2_ samples prepared by the hydrothermal technique do not exhibit a peak around 14°, which indicates poor crystallinity in the (002) oriented plane [25]. Another important aspect is the presence of peaks in the 2θ range between 33° and 59° when MoS_2_ as nanosheets were obtained hydrothermally [25].

**Figure 1:**
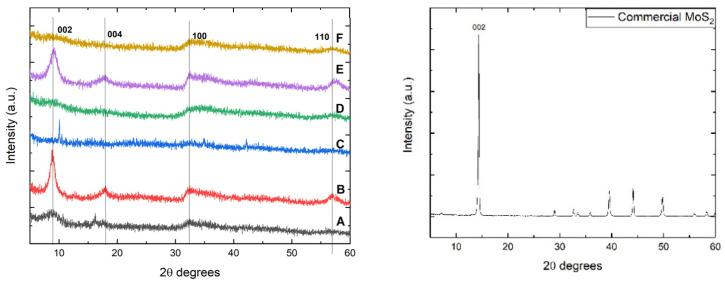
XRD patterns of hydrothermally synthesized MoS_2_ (**left**) and commercial (**right**).

Figure 1 displays the X-ray diffraction patterns of MoS_2_ samples synthesized using different molybdenum and sulfur precursors. It can be observed that samples B and E, synthesized using thiourea as a sulfur source, present the highest crystallinity, as revealed by the peaks at 2θ values of 9°, 18°, 33°, and 58° attributed to (002), (004), (100), and (110) oriented MoS_2_ planes [26].

However, the diffraction peaks attributed to 2θ values of 9° and 18° correspond to (002) and (004) planes of MoS_2_ and are shifted to a lower diffraction angle. These shifts may be attributed to intercalated ammonia between the stacked layers, which originates from the molybdenum precursor. The presence of these two planes also suggests the existence of the metallic 1T-MoS_2_ phase [28,29], in which the Mo and S atoms are shifted from a trigonal prismatic to an octahedral crystal structure.

The sample synthesized using L-cysteine and thioacetamide presents poor crystallinity toward the (002) oriented plane, especially when ammonium molybdate is used (sample D), but it can be observed a minor peak around 2θ values of 33° and 58° degrees, which denotes the presence of a structure with low crystallinity [25]. Sample C, where sodium molybdate and thioacetamide were used, does not exhibit any peak that can be attributed to MoS_2_ planes. By using Bragg’s equation (nλ = 2d sinθ), the interplanar distance of the lattice in the obtained MoS_2_ samples was calculated. The results were in the range of 8.5 to 11.1 Å, corresponding to the metallic crystalline phase of MoS_2_, while for the commercial sample, the result was representative of the 2H semiconductive phase, the distance being 6.16 Å [30]. However, this phenomenon can also be attributed to the XRD patterns of O_2_^−^-incorporated MoS_2_, where the shift of the (002) plane to around 9.30° corresponds to an interlayer distance of 0.95 nm. This shift can be observed regardless of the synthesis temperature in the range of 140–200 °C [31] and will be further discussed in the Raman spectroscopy section to determine the metallic 1T crystalline phase of MoS_2_ or the incorporation of O^2−^ ions between the MoS_2_ layers. The commercial MoS_2_ sample presents a semiconductive crystalline phase with a major peak corresponding to the 002 plane (i.e., 2θ–14°). Typically, oxygen intercalation between MoS_2_ layers leads to an increase in the interlayer spacing, particularly affecting the (002) plane. In standard 2H-MoS_2_, this plane exhibits a diffraction peak around 14.4°, corresponding to a d-spacing of approximately 6.2 Å. When oxygen is intercalated, the peak shifts to lower angles, around 9.3°, indicating an expanded interplanar distance of about 9.5 Å [30,31]. This expansion is likely due to the insertion of O^2−^ ions or Mo–O species between the S–Mo–S layers, which can disrupt the van der Waals interactions and potentially stabilize a metastable phase, such as the metallic 1T phase. In the presented XRD diffractograms, samples A, B, and E exhibit characteristic peaks corresponding to the (002), (004), (100), and (110) planes of MoS_2_. The noticeable shift of the (002) peak to lower angles in these samples may be attributed to the increased oxygen content observed in the EDX spectra, suggesting greater interlayer spacing. A similar phenomenon was reported by Silambarasan et al., where MoS_2_ was intercalated with CTA^−^ ions, resulting in interlayer expansion [32].

The average crystallite size was determined using the Scherrer equation and taking into account the value of the Full Width at Half Maximum (FWHM). For the sample utilizing L-cysteine as a sulfur source and sodium molybdate, this value was found to be 1.9 nm; meanwhile, for sample D, the average crystallite size could not be calculated. An increase in the average crystallite size was obtained for samples B and E, namely 8.16 and 5.62 nm, respectively. The crystallite mean size obtained for sample C, which was prepared using sodium molybdate and thioacetamide, was 82.45 nm, while for sample F, the FWHM and mean crystallite size could not be determined.

**Figure 2:**
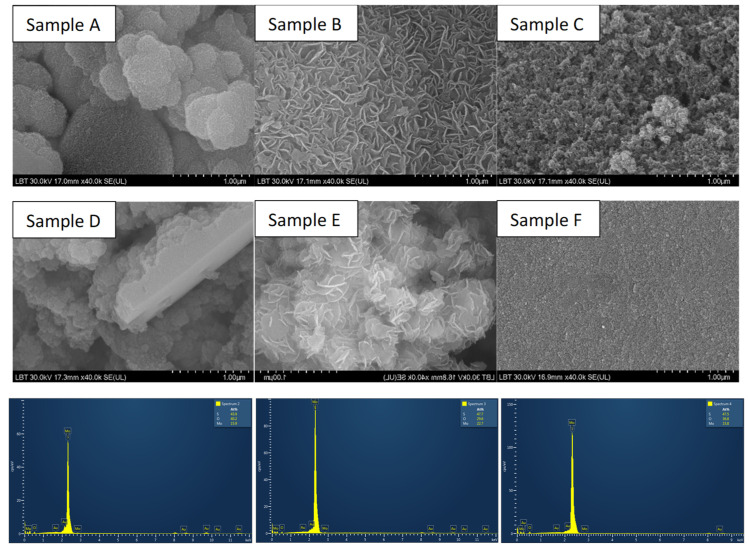

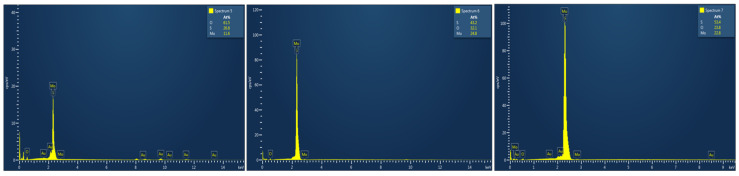
SEM images of MoS_2_ samples using sodium molybdate and L-cysteine (Sample A), thiourea (Sample B), thioacetamide (Sample C), and ammonium molybdate combined with L-cysteine (Sample D), thiourea (Sample E), thioacetamide (Sample F), and their corresponding EDX measurements (presented in the same order as the samples in the SEM images).

The morphology and elemental composition of MoS_2_ samples were identified by SEM and EDX (Figure 2 and Table 2). The images show typical MoS_2_ morphologies. It can be seen that many irregular nanosheets aggregate together and assemble into flower-like microsphere structures besides sample F, which shows a smooth surface where there is no sign of formed nanosheets. From the SEM images, it can be observed that there are similarities between samples A–D and B–E. Samples synthesized with L-cysteine as the sulfur precursor (samples A and D) exhibit a sphere-like morphology, in contrast to the flower-like morphology observed in previous studies. On the other hand, samples prepared with thiourea (samples B and E) show larger aggregated nanosheets that tend to form the typical MoS_2_ flower-like structure [33]. Sample C, prepared using thioacetamide (TAA) and sodium molybdate, exhibits smaller aggregates that do not conform to a specific MoS_2_ flower-like or sphere-like morphology. This can be attributed to the formation of MoS_3_ due to the use of these precursors [34], which is further confirmed by EDX analysis showing a Mo to S ratio of 1:3 (see Table 2).

The sample prepared using sodium molybdate and thiourea shows an atomic ratio of 1:2.10, which is close to the stoichiometry of MoS_2_. All samples, except for the one using ammonium molybdate and thiourea, show an increase in sulfur content. This can be attributed to the excess sulfur used in the synthesis process to enhance crystallinity.

**Figure 3:**
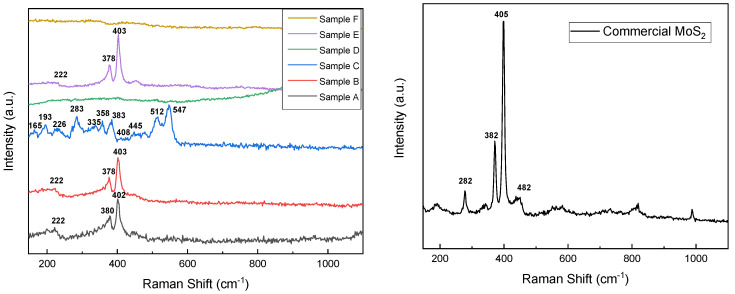
Raman spectra of MoS_2_ as-prepared samples (**left**) and commercial samples (**right**).

To investigate the vibrational energy states of obtained MoS_2_ nanomaterials, Raman spectra have been recorded and presented in Figure 3. The commercial sample, along with samples A, B, and E, exhibits typical MoS_2_ spectra with its characteristic peaks E_2_g^1^ and A_1_g at 378 cm^−1^ for sample A and 380 cm^−1^ for samples B and E, respectively, at 402 cm^−1^ and 403 cm^−1^ for samples A, B, and E. E_2_g^1^ mode is ascribed to in-plane vibration of sulfur atoms toward Mo in the tri-layer of MoS_2_ and A_1_g mode is attributed to out-of-plane vibration of sulfur atoms in the opposite direction [35]. The commercial sample appears to exhibit characteristics of the 2H semiconductive crystalline phase of molybdenum disulfide, whereas the as-prepared samples (A, B, and E) display characteristics of the metallic 1T phase. It can be observed that the frequency of A_1_g mode decreases (red shift) and E_2_g^1^ mode increases (blue shift) for sample A, which can be attributed to long-range interlayer coulombic interactions in multilayer MoS_2_ or stacking-induced structure changes may dominate the charge of atomic vibrations rather than interlayer van der Waals forces [36]. Samples A, B, and E also exhibit a peak at 220 cm^−1^, corresponding to the 1T metallic crystalline phase of MoS_2_, which was further confirmed by XRD measurements [37]. Sample C presents peaks around 160 and 220–230 cm^−1^ (corresponding to the Mo-S in-plane bending modes), 310–390 and 450 cm^−1^ for the Mo-S stretching modes, and 520–555 cm^−1^ (S-S stretching modes) of MoS_3_, ref. [28] result that was confirmed by the elemental composition ratio obtained from EDX. One should mention that samples D and F did not present any signals related to MoS_2_.

**Figure 4:**
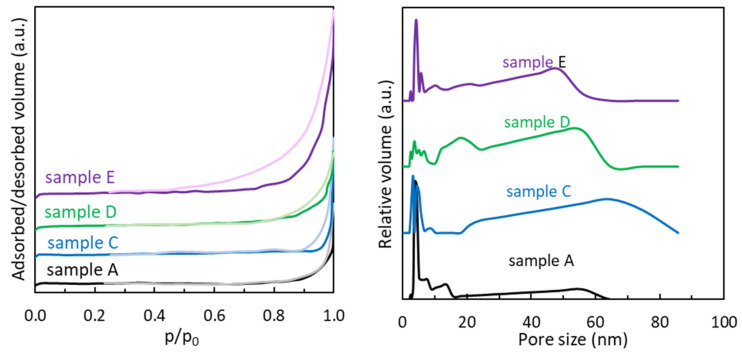
N_2_ adsorption/desorption isotherms (**left**) and pore size distribution (**right**) of MoS_2_ samples synthesized at 200 °C for 24 h.

The N_2_ adsorption–desorption isotherms of the MoS_2_ samples (Figure 4 left) are similar in shape, being of type IV according to IUPAC classification [28], indicating the presence of mesopores, which can also be seen in the pore size distribution plots presented in Figure 4, right. The hysteresis loops are of type H3, more pronounced for sample E and gradually decreasing in amplitude for samples D, C, and A. This type of hysteresis is usually associated with the presence of plate-like aggregates, which is consistent with SEM images for MoS_2_ samples (Figure 2), but also with the presence of partially filled macropores, which can also be observed in Figure 4, right.

Surface area and pore structure features of MoS_2_ were calculated from the nitrogen adsorption–desorption isotherms data. The specific surface area of MoS_2_ samples (Table 3) varies with the molybdenum and sulfur precursor. For all samples, the surface area is higher than that obtained for the commercial sample (1.5 m^2^/g). For both molybdenum precursors, the samples prepared from L-cysteine have lower surface areas than those prepared from other sulfur precursors. Also, for all sulfur precursors, the samples derived from ammonium molybdate have a higher surface area than those obtained from sodium molybdate.

The pore size distribution plots of MoS_2_ presented in Table 3 and Figure 4 (right) reveal multimodal pore size materials for all samples, with two similar main regions of pores size: small mesopores in the narrow 3–10 nm region and a wide region of large mesopores–small macropores (20–60 nm for samples A, D, and E and 20–80 nm for sample C). The difference in surface area can be attributed to the different morphologies obtained using various precursors. However, pore size does not seem to be very different, which can potentially be due to the hydrothermal approach, even if the precursors are different.

Wang et al. [29] reported a BET surface area of flower-like MoS_2_ of 16.68 m^2^/g with a size of the average pore width of 16.8 nm that led to a specific capacitance of 168 F·g^−1^ at a current density of 1 A·g^−1^. Singh et al. [38] used ammonium hepta-molybdate tetrahydrate and thiourea to synthesize flower-like MoS_2_ for photolytic applications and SERS sensing and reported surface areas between 5 and 20 m^2^/g, which can be related to our value when using the same precursors. Sun et al. [39] synthesized a MoS_2_-graphene composite using sodium molybdate and thiourea alongside graphene oxide (GO) and obtained a specific capacitance of 168 F·g^−1^ at a current density of 1 A·g^−1^ with a specific surface area of 165.7 m^2^/g due to the addition of GO. However, it can be seen from their pore size distribution that the pore volume of pristine MoS_2_ is significantly lower than that of the reported GO-doped MoS_2_.

### 3.2. Electrochemical Characterization

**Figure 5:**
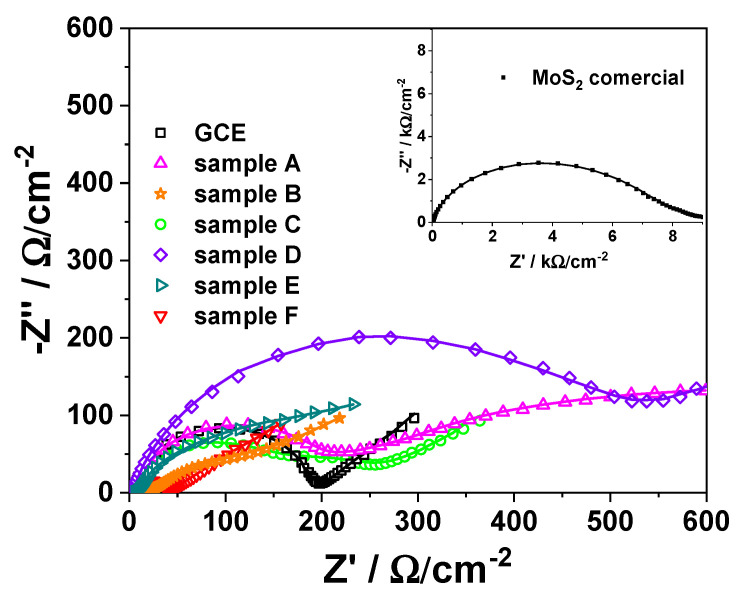
Nyquist plots recorded at bare GCE and modified GCE based on MoS_2_ samples A-F and at commercial MoS_2_-based modified electrode (inset). Experimental conditions: electrolyte, 0.1 M KCl solution containing 5 mM K_4_[Fe(CN)_6_]/K_3_[Fe(CN)_6_]; frequency range, 10^−2^–10^4^ Hz; amplitude, 10 mV; time for OCP measurements, 3600 s; fitted data (solid lines).

Electrochemical characterization of the interfacial properties of the MoS_2_-based modified electrodes was realized by electrochemical impedance spectroscopy (EIS) technique [40] in [Fe(CN)_6_]^3−/4−^ electrochemical probe. The EIS records were fitted to a modified Randless equivalent circuit [40,41] R_el_(Q_f_(R_f_(Q_dl_(R_ct_W)))) for all the synthesized MoS_2_-based modified electrodes, R_el_(Q_f_(R_f_(Q_dl_(R_ct_)))) for the commercial MoS_2_-based modified electrodes, and R_el_(Q_dl_(R_ct_W)) for bare GCE, respectively (Figure 5). The proposed circuit consists of an electrolyte solution resistance (R_el_) combined in series with a parallel grouping of the MoS_2_ layer electrolyte interface, apparent capacitance, Q_f_, and layer resistance, R_f_, which in turn is connected in series with a parallel arrangement consisting in a pseudo-capacitive charge storage (Q_dl_) and a charge transfer resistance (R_ct_)]. Finite Warburg impedance W connected in series to R_ct_ was considered for ions diffusion through the pores of materials.

The values of all the above-mentioned parameters were estimated (Table 4) and were used to compare the six synthesized electrode materials (A–F) and to get further to their potential applications.

As can be seen from the obtained chi^2^ values (Table 4) lower than 10^−3^, it can be concluded that there is a very good correlation between the experimental and theoretical data, which confirms the right choice of the equivalent circuit. The charge transfer resistance (R_ct_) values decrease significantly for the synthesized samples in comparison with the commercial one (from one order of magnitude if compared to sample D to more than 2 orders of magnitude (140 times) if compared with sample F), as reflected in the following sequence: F < C < B < CGC < E < A < D < commercial. This means that at the modified electrode based on MoS_2_ synthesized materials (A–F) the charge transfer is easier in comparison with the commercial one. Interestingly, the R_ct_ values lower than those obtained for bare GCE lead to the conclusion that samples F, C, and B can work as good electrode modifiers (in this order), which evidence possible catalytic properties for modified electrodes developing for electroanalytical applications (electrochemical sensors development).

At the same time, as expected, the morpho-structural parameters (porosity, crystallinity, etc.) of the MoS_2_ matrix strongly influence the material capacitance (Q_f_), but rather the material’s crystallinity, as reflected in the following sequence: F < D < C < commercial < A < B < E. Samples F, D, and C, respectively, show low crystallinity, which is well reflected in the electrochemical performances. Thus, the samples with high crystallinity A, B, and E (B and E showing the highest peak associated with the (002) plane that corresponds to a typical MoS_2_ structure) show higher capacitance and can be used in supercapacitor development. Among these, E has the highest capacitance. This fact indicates that, depending on the focused application (supercapacitors or electrochemical sensors), the precursors used in synthesis play a key role. The obtained results correlate well with those observed by Cheng et al. for CuCo_2_S_4_ composite [42]. In the obtained series, the commercial MoS_2_ is situated in the middle, which proves that the material’s electrochemical properties are strongly correlated with the morpho-structural parameters (specific surface area, pore size, crystallinity, etc.), and all these corroborated lead to the final material performance.

It must be noticed that in the region corresponding to lower frequencies (Figure 5), attributed to the diffusion-controlled processes, the W values at GCE, C, B, and E modified electrodes show a similar behavior (GCE ≈ C ≈ B ≈ E). The following sequence, GCE ≈ C ≈ B ≈ E < F < A, was obtained (Table 4). The increase in the W value indicates an acceleration of the diffusion rate due to the corroborated effect of the material’s properties, such as porosity. Thus, it can be observed from the sorption measurement results (Table 3) and EIS data (Table 4) that the prepared MoS_2_ samples (i.e., A–F) have higher porosity (i.e., the total pores volume and the pore size) and higher W values, in comparison with the commercial one. On the other hand, it can be observed that sample A, which has the lowest surface area (1.5 m^2^/g), exhibits the best capacitive behavior, achieving a capacitance of 29 mF/cm^2^ without the addition of any conductive template such as rGO or graphene. However, for the samples with higher surface areas, the capacitance appears to be lower, according to the results obtained from the EIS measurements. Thus, taking into account all these aspects related to the sorption measurements (i.e., specific surface area and pore values) and the electrochemical data closely linked to the diffusion process, one can infer that the pores and their characteristics play a key role in improving electrochemical performance.

The synergistic effects of the MoS_2_ morpho-structural parameters (size, porosity, crystallinity), which are the results of the differences in the synthesis procedure of samples A–F, can effectively improve the ion diffusion dynamics and the conductivity of the electrode materials, which can greatly enhance the material’s performances for a focused application: supercapacitors or sensing platform.

The calculated pseudo-capacitance values indicate that for all MoS_2_ synthesized samples (A–F), enhanced values were obtained in comparison to the commercial one. As mentioned above, the electrochemical properties of hydrothermally synthesized MoS_2_ can be directly correlated with a series of morpho-structural properties, which can also be correlated with synthesis parameters and precursor types used in the reaction. Therefore, one needs to find the perfect balance between the precursor types and hydrothermal reaction parameters to obtain a homogeneous MoS_2_ powder with high crystallinity, a large surface area, and promising morphology. The corroborated morpho-structural properties directly influence the electrochemical ones. In this study, the most promising materials were samples B and E, which were synthesized using different Mo precursors but with the same sulfur source (thiourea). It can also be observed that samples B and E, which were synthesized using different Mo precursors, but with the same sulfur source (thiourea), possess higher crystallinity than other synthesized samples and higher specific surface area, and they tend to form flower-like morphologies, based on many self-assembled flakes, with greater flake interspacing. Taking all of this into account, it can be seen from the electrochemical parameters’ values (Table 3) that by hydrothermal synthesis of MoS_2_ using thiourea in excess as a sulfur source, materials with better morpho-structural and electrochemical properties can be obtained.

## 4. Conclusions

The present study highlighted the differences that can appear in MoS_2_ performances when hydrothermal procedure synthesis is used and different precursors for molybdenum sources (ammonium heptamolybdate tetrahydrate (and sodium molybdate hydrate) and sulfur sources (L-cysteine, thiourea, and thioacetamide) are involved. The six samples A–F were morphologically and structurally characterized by XRD, Raman spectroscopy, N_2_ adsorption/desorption measurements, and SEM-EDX. The electrochemical impedance spectroscopy measurements were performed on all obtained samples by evaluating the electrochemical performance of a MoS_2_-based electrode material. The most promising sample in our study for supercapacitor applications is sample A (using sodium molybdate dihydrate and L-cysteine as precursors) with a surface area of 1.5 m^2^/g, obtaining a capacitance of 29 mF/cm^2^. However, in terms of crystallinity and morphology, samples using thiourea as the sulfur source yielded the best results by having the highest crystallite size, 8.16 nm for sample B and 5.62 nm for sample E, as well as the flower-like morphology, even if they present lower capacitance from EIS measurements.

A better understanding and correlation of the precursor type used in the synthesis reaction with the morpho-structural properties and the electrochemical performances will lead to finding appropriate applications (such as supercapacitors, batteries, photovoltaics, catalytic, and sensing). Accordingly, a low charge transfer resistance value obtained for samples F, C, and B was correlated with good catalyst properties for sensor development for electroanalytical applications. On the other hand, a high capacitance value obtained for samples E, B, and A was correlated with the crystallinity increase, and such materials are recommended in supercapacitors applications.

## Figures and Tables

**Table 1:** Samples prepared using different sources of molybdenum and sulfur.

Molybdenum Precursor (42 mM)	Sulfur Precursor (840 mM)	Molar Ratio of Mo: S Precursors	Sample Name
Na_2_MoO_4_·2H_2_O	C_3_H_7_NO_2_S	1:20	A
Na_2_MoO_4_·2H_2_O	CH_4_N_2_S		B
Na_2_MoO_4_·2H_2_O	C_2_H_5_NS		C
(NH_4_)_6_Mo_7_O_24_·4H_2_O	C_3_H_7_NO_2_S		D
(NH_4_)_6_Mo_7_O_24_·4H_2_O	CH_4_N_2_S		E
(NH_4_)_6_Mo_7_O_24_·4H_2_O	C_2_H_5_NS		F

**Table 2:** Elemental composition and Mo to S ratio of MoS_2_ samples as determined from the EDX measurements.

Sample Name	Elemental Composition (Atomic %)			Mo:S Ratio	Incorporated O^2−^ (Atomic %)
	Mo	S	O		
A	15.9	43.9	40.2	1:2.76	40.2
B	22.7	47.7	29.6	1:2.10	29.6
C	15.8	47.5	36.8	1:3.00	36.8
D	11.6	26.8	61.5	1:2.31	61.5
E	24.8	43.2	32.1	1:1.74	32.1
F	22.8	53.4	24.8	1:2.34	24.8

**Table 3:** Surface area and porosity parameters of the MoS_2_ materials obtained using different molybdenum and sulfur precursors (BET surface area, total pore volume, and pore diameter).

Sample	S_t_ (m^2^/g)	V_p_ (cm^3^/g)	D_m_ (nm)
Commercial	1.5	0.03	-
A	8	0.07	4–15; 20–60
C	18	0.10	4–10; 20–80
D	20	0.10	4–10; 10–65
E	28	0.11	4–10; 20–60

**Table 4:** The parameters of the equivalent circuit.

Electrode	A	B	C	D
**R_el_ (Ω × cm^2^)**	5.48 ± 0.26	6.69 ± 2.77	4.70 ± 0.81	4.01 ± 2.85
**Q_f_ (µS × s^n^/cm^2^)**	39.54 ± 0.79	54.27 ± 15.91	25.00 ± 3.05	19.94 ± 4.18
**n**	0.91	0.76	0.89	0.86
**R_f_ (Ω × cm^2^)**	145.0 ± 0.21	17.06 ± 3.08	141.5 ± 2.25	477.4 ± 3.07
**Q_dl_ (mS × s^n^/cm^2^)**	13.34 ± 0.57	5.39 ± 2.57	1.51 ± 6.27	2.13 ± 6.22
**n**	0.74 ± 0.75	0.557 ± 1.65	0.600 ± 4.66	0.52 ± 9.76
**R_ct_ (Ω × cm^2^)**	694.3 ± 4.05	158.6 ± 3.86	127.5 ± 4.95	971.9 ± 19.19
**W (mS × s^1/2^/cm^2^)**	230.5 ± 12.27	30.08 ± 2.59	28.92 ± 2.09	-
**C (µF/cm^2^)**	29,160	4760	500	4170
**χ^2^**	0.0000314	0.0001655	0.000114	0.0008212
				
**Electrode**	**E**	**F**	**commercial**	**GCE**
**R_el_ (Ω × cm^2^)**	4.82 ± 6.79	4.41 ± 1.14	7.66 ± 1.80	4.81 ± 0.57
**Q_f_ (µS × s^n^/cm^2^)**	1845 ± 8.18	19.06 ± 6.15	14.97 ± 4.34	
**n**	0.56	0.92	0.292	
**R_f_ (Ω × cm^2^)**	4.29 ± 20.31	32.1 ± 2.23	13.42 ± 1.044	-
**Q_dl_ (mS × s^n^/cm^2^)**	6.57 ± 17.62	16.79 ± 9.10	1.79 ± 5.44	13.02 ± 1.15
**n**	0.765 ± 3.97	0.407 ± 2.80	0.91 ± 1.20	0.92 ± 0.14
**R_ct_ (Ω × cm^2^)**	225.9 ± 3.04	69.6 ± 5.31	9763 ± 0.78	186.5 ± 0.19
**W (mS × s^1/2^/cm^2^)**	31.67 ± 13.53	57.59 ± 2.58	-	27.71 ± 0.85
**C (µF/cm^2^)**	7420	21070	1.2	7.71
**χ^2^**	0.0003559	0.000115	0.00004114	0.000066

(±) represents the relative standard deviation expressed in %.

## Data Availability

The original contributions presented in the study are included in the article, further inquiries can be directed to the corresponding authors.
